# Racial and Ethnic Disparities in the Burden and Cost of Diabetes for US Medicare Beneficiaries

**DOI:** 10.1089/heq.2019.0004

**Published:** 2019-05-15

**Authors:** Namino M. Glantz, Ian Duncan, Tamim Ahmed, Ludi Fan, Beverly L. Reed, Samaneh Kalirai, David Kerr

**Affiliations:** ^1^ Sansum Diabetes Research Institute, Santa Barbara, California.; ^2^ Department of Statistics and Applied Probability, University of California Santa Barbara, Santa Barbara, California.; ^3^ Santa Barbara Actuaries, Inc., Santa Barbara, California.; ^4^ Eli Lilly and Company, Indianapolis, Indiana.

**Keywords:** burden of diabetes, elderly, health care, Hispanic population, Medicare, public health

## Abstract

**Purpose:** To examine the burden and cost of diabetes among fee-for-service Medicare beneficiaries.

**Methods:** Medicare 5% File data for type 1 diabetes (T1D) and type 2 diabetes (T2D) consisting of 1,397,933 enrollees in fee-for-service without Medicare Advantage during the period 2012–2013 were analyzed by race and ethnicity.

**Results:** Although non-Hispanic whites (nHWs) comprised most of this population (86%), prevalence of T1D and T2D was higher for Hispanics than nHWs (3.4% vs. 1.8%, *p*=0.0006, for T1D and 33.4% vs. 21.9%, *p*<0.0001, for T2D). Hispanics also had more acute hospital admissions (*p*=0.0235 for T1D and *p*=0.0009 for T2D) and longer lengths of stay (7.5 vs. 6.9 days for T1D, *p*=0.0105, and 6.7 vs. 6.2 days for T2D, *p*<0.0001) compared with nHWs. Allowed and paid costs per member per month adjusted for confounding were higher for Hispanics than nHWs for T2D (both *p*<0.0001) and lower for those with T1D (both *p*<0.0001). Mean number of chronic diseases in patients with diabetes was higher in Hispanics than nHWs (both T1D and T2D, *p*<0.0000). For T2D, Hispanics were more likely to have glycated hemoglobin (HbA_1c_) and lipid testing as well as nephropathy screening (all *p*<0.0001). Hispanics with T1D were also more likely to have HbA_1c_ and lipid tests (*p*=0.0014 and *p*=0.0011, respectively); retinopathy and nephropathy screening rates did not differ significantly from rates among nHWs.

**Conclusion:** Diabetes disproportionately impacts US seniors, with Hispanics almost twice as likely as nHWs to be diagnosed. Racial and ethnic disparities exist in the burden and cost of diabetes care for Medicare recipients.

## Introduction

In the United States, the rise in prevalence of diabetes, especially type 2 diabetes (T2D), has been particularly marked within racial and ethnic minority populations and among older individuals.^1,2^ This increase in the number of individuals developing diabetes has added a significant financial burden to health care systems with current annual global costs estimated to be US $673 billion and these are projected to rise to US $802 billion by 2040.^3^ In the United States, diabetes care already accounts for one of every four dollars spent on health care.^4^

Both old age and diabetes are important risk factors for functional decline and disability in older people with the potential to add significant costs to health care systems.^5^ As a corollary, being uninsured or a Medicaid recipient presents formidable challenges to improving cost-effective outcomes for people with diabetes.^6,7^ Currently, in the United States, more than 29 million people are uninsured, with substantial inequalities in access to health care along economic, gender, racial, and ethnic lines.^8^ Previous studies have documented that racial and ethnic minority groups also receive low quality of health care, including preventative health services, compared with their white counterparts^9–11^ and that racial and ethnic minority groups have higher rates of diabetes-related complications.^12–17^

More recently, there is evidence that for Medicare beneficiaries in managed care plans (including Medicare Managed Care and Medicare Fee-for-Service plans) as well as for Veterans Administration populations, there has been progress in reducing disparities in diabetes care.^18,19^ However, it remains to be determined whether similar improvements have been seen in other US populations based on race and ethnicity and what impact they have had on the economic costs of diabetes.

While old age and diabetes place a disproportionate burden on racial and ethnic minorities compared with non-Hispanic whites (nHWs), Hispanics (who comprise 18% of the US population) are almost twice as likely to develop T2D as nHWs and experience high rates of poorly controlled diabetes and related complications.^20–23^ Therefore, the aim of this study was to identify differences in diabetes prevalence, health care resource utilization, and cost for Medicare beneficiaries among Hispanics compared with nHWs with both T2D and type 1 diabetes (T1D).

## Methods

This retrospective analysis used data from the Medicare Limited Data Set (Medicare 5% File) consisting of fee-for-service Medicare enrollees (∼5% of all Medicare beneficiaries) for the years 2012–2013. Participants were excluded if they had Medicare Advantage coverage, had fewer than 6 months of Medicare eligibility, did not have both Medicare fee-for-service Parts A and B coverage, or were aged below 65 years, but had Medicare coverage because of permanent disability.

For the purposes of analyses, in the Medicare 5% File, Hispanic is defined directly as a single variable rather than as separate race and ethnicity variables. This definition ignores the two variables usually used to define Hispanics—race and ethnicity. The term Hispanic refers to people who share Spanish as their common language and this is the nomenclature used by Medicare to describe enrollees' self-perception of race and ethnicity. This may have resulted in underrepresentation of Hispanics in the Medicare 5% File.^24,25^

### Statistical analysis

To test whether there are differences in the prevalence of T1D and T2D by race and ethnicity among different age groups and genders, chi-square tests were conducted separately for T1D and T2D populations. SAS (SAS Institute, Cary, NY) was used to perform the analysis.

To test for differences in mean allowed and paid amounts per member per month (PMPM) between racial and ethnic groups and between T1D and T2D, *z*-tests were conducted.

To test whether costs for both allowed and paid amounts PMPM differed by racial and ethnic groups for T1D and (separately) for T2D, the Wilcoxon rank-sum test was used as the sample size was unequal for nHW and Hispanic populations.

Allowed amount (charge) was defined as submitted charges less any discounts that apply and ineligible charges. Allowed charges were calculated before any patient cost sharing was applied; after patient cost sharing, charges are referred to as net paid claims. Paid amount was defined as the allowed amount less beneficiary responsibilities (deductible and coinsurance, etc.).

Adjustments using regression models or propensity scores were used to minimize potential confounders of the relationship between race and ethnicity and PMPM cost. We applied both these approaches to each of T1D, T2D, and individuals without diabetes, comparing outcomes in Hispanic and nHWs. All models were analyzed for Hispanic and nHWs, for patients with T1D and T2D, and for years 2012–2013 combined. To analyze variations in total allowed and paid amounts PMPM, we compared cost variations between Hispanic versus white groups using a general linear model.

Risk scores were calculated using the Centers for Medicare & Medicaid Services (CMS) hierarchical condition categories (HCCs) (Version 22).^26^ The HCC model is a method for assigning a single relative measure of relative risk and is used for calculating relative risk for transfer payments among Medicare Advantage health plans. The type and number of chronic conditions are calculated using the CMS definition of chronic conditions. HCCs are an example of so-called grouper models that group individual International Classification of Diseases (ICD)-9 and ICD-10 codes into categories of like diagnoses. These groups simplify the task of analyzing data by diagnosis, while at the same time providing regular updates to their underlying algorithms. The model results in an estimate of the relative risk (combined with an underlying average cost for a group of people with diabetes) that allows analysts to predict the relative cost for each individual. Coefficients of the regression model provide an estimate of the relative contribution of each category of HCCs to the individual's predicted cost.

## Results

A total of 1,397,933 individuals who met the eligibility criteria were included in the study. Racial and ethnic distribution of the sample showed that 1.7% were Hispanic, 1.9% were Asian, 7.8% were black, 86.1% were nHWs, and 2.5% were other.

### Prevalence of T1D and T2D

The prevalence of both T1D and T2D was significantly higher in Hispanics compared with nHWs (*p*<0.0006 and *p*<0.0001, respectively) (Table 1).

**Table 1.** Prevalence of Type 1 and Type 2 Diabetes in Individuals Stratified by Age, Racial and Ethnic Group, and Gender from the 2012 to 2013 Medicare 5% Sample

	**Hispanic (H) (%)**	**Non-Hispanic Whites (nHWs) (%)**	***p*-Value**
Age (years)			
Type 1 diabetes			
65–69	1.8	1.1	0.0000
70–74	2.9	1.4	0.0000
75–79	4.2	2.7	0.0000
80–84	5.1	2.4	0.0000
85–90	4.5	2.2	0.0000
>90	4.7	1.6	0.0000
Total	3.4	1.8	0.0000
Type 2 diabetes			
65–69	25.8	17.0	0.0000
70–74	31.0	20.6	0.0000
75–79	38.8	27.5	0.0000
80–84	40.3	26.2	0.0000
85–90	39.2	24.2	0.0000
>90	34.9	19.6	0.0000
Total	33.4	21.9	0.0000
Sex			
Type 1 diabetes			
Male	39.2	46.6	0.0089
Female	60.8	53.4	0.0012
Type 2 diabetes			
Male	39.6	47.1	0.0000
Female	60.4	52.9	0.0000

There were significant differences by race and ethnicity for gender distribution, with higher rates of T1D and T2D in women than men in both Hispanic and nHW populations, a difference that is more skewed in Hispanics than nHWs. Specifically, 60.8% of Hispanics with T1D are female, while 53.4% of nHWs with T1D are female (*p*=0.0012). Likewise, 60.4% of Hispanics with T2D are female, while 52.9% of nHWs with T2D are female (*p*=0.0000). The prevalence of both T1D and T2D in various age groups also differed significantly between Hispanics and nHWs (*p*<0.0001) (Table 1).

The prevalence of both T1D and T2D increased with age among nHWs, with the highest rate noted in individuals aged 75–79 years, after which the prevalence gradually declined for both T1D and T2D. However, in Hispanics, the highest prevalence of T1D and T2D was observed in the age group of 80–84 years.

### Costs PMPM

The overall allowed and paid costs PMPM for individuals with and without diabetes and Hispanics versus nHWs are shown in Table 2 (unadjusted and adjusted). Overall, both the allowed and paid adjusted and unadjusted costs PMPM were two- to fourfold higher for individuals with diabetes compared with those without (*p*<0.0001).

**Table 2.** Unadjusted and Adjusted Average (Mean) Costs (Allowed and Paid) Per Member Per Month by Type of Diabetes and Racial and Ethnic Group (Hispanics Compared with Non-Hispanic Whites) from the 2012 to 2013 Medicare 5% Sample

**Type of diabetes**		**Average amount per member per month**						**Difference: Hispanics vs. nHWs**	
	**Race**	**2012**	**2013**	**2012**	**2013**	**2012–2013^a^**		**2012–2013^a^**	
		**Allowed ($)**		**Paid ($)**		**Allowed ($)**	**Paid ($)**	**Allowed**	**Paid**
								***p*^a^**	
**Unadjusted**									
No diabetes	Hispanic	461	424	399	367	442	383	<0.0001	<0.0001
No diabetes	nHW	557	548	477	468	552	472		
T1D	Hispanic	2708	2747	2354	2381	2726	2366	<0.0001	<0.0001
T1D	nHW	2223	2191	1928	1890	2208	1910		
T2D	Hispanic	1390	1431	1211	1249	1410	1230	<0.0001	<0.0001
T2D	nHW	1233	1241	1067	1071	1237	1069		
**Adjusted**									
No diabetes	Hispanic	608	643	530	565	626	548	<0.0001	<0.0001
No diabetes	nHW	528	616	451	529	572	490		
T1D	Hispanic	2138	2292	1869	1999	2214	1933	<0.0001	<0.0001
T1D	nHW	2176	2479	1886	2148	2320	2010		
T2D	Hispanic	1170	1468	1020	1294	1320	1158	<0.0001	<0.0001
T2D	nHW	1214	1387	1050	1203	1300	1127		

^a^ 2012–2013 average.

T1D, type 1 diabetes; T2D, type 2 diabetes; nHW, non-Hispanic white.

For individuals with T2D, unadjusted and adjusted allowed and paid costs PMPM for 2012–2013 combined were higher for Hispanics compared with their nHW counterparts (both *p*<0.0001). For individuals with T1D, unadjusted costs were higher for Hispanics, but after adjusting for potential confounders, costs were higher for nHWs.

### Resource utilization

**FIG. 1.**
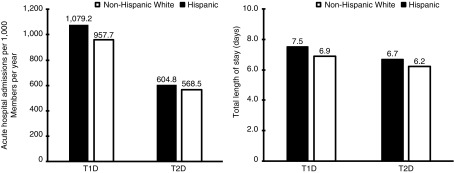
Resource utilization: acute hospital admissions per 1000 members per year and total length of stay (days) by type of diabetes and racial and ethnic group from the 2012 to 2013 Medicare 5% sample.

There was no significant difference in total admissions between Hispanics and nHWs with T1D. However, the distribution of admissions was different for the two groups, with Hispanics having significantly more acute hospital admissions than nHWs (1079 in Hispanics vs. 958 in nHW admissions per 1000 members per year; *p*=0.0235). Total admissions among people with T2D were lower than for people with T1D for both Hispanics and nHWs. Acute hospital admissions were significantly higher for Hispanics with T2D compared with nHWs with T2D (605 in Hispanics vs. 569 in nHW admissions per 1000 members per year, *p*=0.0009). The length of stay during acute hospital admissions was significantly longer for Hispanics compared with nHWs with both T1D (7.5 in Hispanics vs. 6.9 in nHWs, *p*=0.0105) and T2D (6.7 in Hispanics vs. 6.2 in nHWs, *p*<0.0000) (Fig. 1). There were no significant differences in the use of services such as office visits, specialist visits, and surgery rates between Hispanics and nHWs with T1D. However, nHWs with T2D had significantly more office visits and minor surgeries per year than Hispanics with T2D (*p*=0.0336 and *p*<0.0000, respectively).

### Burden of chronic disease

**FIG. 2.**
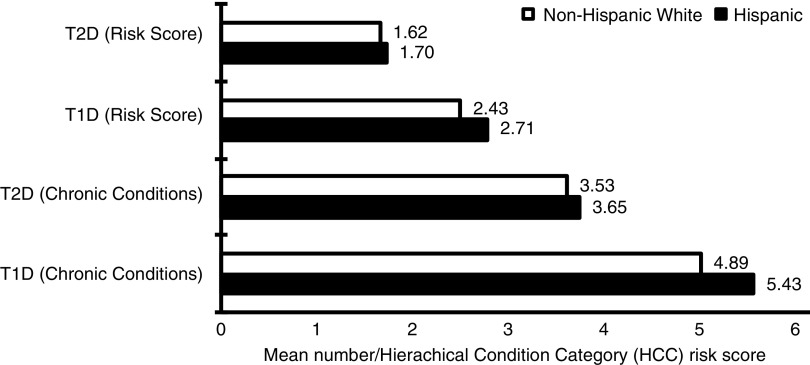
Relative risk and average number of chronic conditions among Hispanic versus non-Hispanic white populations from the 2012 to 2013 Medicare 5% sample. T1D, type 1 diabetes; T2D, type 2 diabetes.

The relative risk and average number of chronic conditions were significantly higher in the Hispanic population compared with nHWs in both patients with T1D and T2D (*p*<0.0000) (Fig. 2). The higher incidence of chronic conditions is reflected in the relative HCC risk score since chronic conditions are the basis of the risk score. Notably, compared with nHWs with diabetes, Hispanics with either T1D (3.9% vs. 9.0%, respectively) or T2D (1.4% vs. 4.2%, respectively) had a much higher prevalence of end-stage renal disease (ESRD) (both *p*<0.0000). A comparison of costs showed that the allowed amount (claims less discounts/ineligible charges) for treating ESRD was higher for Hispanics with T1D than nHWs with T1D ($8,946 vs. $8,332 PMPM, respectively, *p*<0.0000). In contrast to other racial and ethnic groups, it did not cost significantly more to treat Hispanics with T2D who had ESRD compared with nHWs with T2D who had ESRD (allowed costs $6,634 vs. $6,542, respectively, *p*=0.6931). Similar results were observed for net paid costs, taking into account cost sharing. Average cost sharing for all patients with ESRD was $730 (no diabetes), $1177 (T1D), and $939 (T2D) PMPM, respectively.

### Quality metrics

**FIG. 3.**
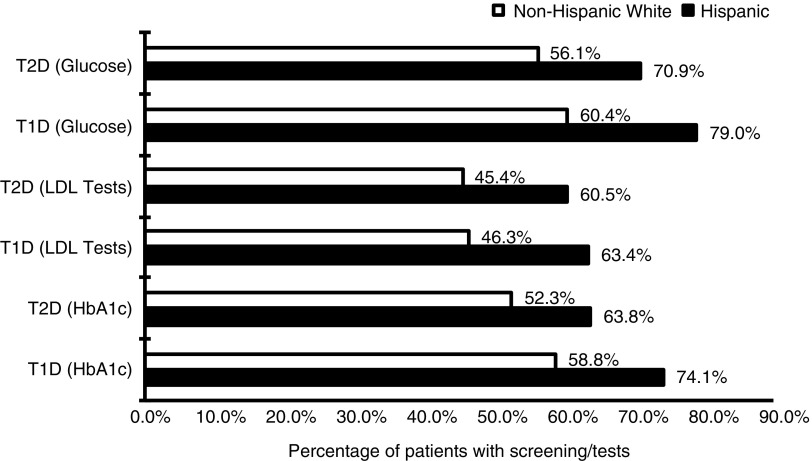
Reported rates for glucose, low-density lipoprotein, and glycated hemoglobin measurements by type of diabetes and race from the 2012 to 2013 Medicare 5% sample. HbA1c, glycated hemoglobin; LDL, low-density lipoprotein.

Hispanics with T1D were significantly more likely to have glucose, lipid (low-density lipoprotein cholesterol), and glycated hemoglobin (HbA_1c_) tests than nHWs with T1D (*p*<0.001) (Fig. 3). Hispanics with T2D also had significantly higher rates of HbA_1c_, lipid, and nephropathy screening tests (all *p*<0.0001).

## Discussion

In the United States, Hispanics represent the fastest growing ethnic and minority group and are known to have an excess burden of diabetes and associated complications compared with the general population.^27^ Meanwhile, the number of older people developing diabetes has also increased exponentially over recent years.^28^ It is also known that older people with diabetes have significantly more comorbidities, such as myocardial infarction, stroke, peripheral arterial disease, and renal impairment, compared with older people without diabetes.^2^ To assess the burden of diabetes among seniors by race and ethnicity in the United States, we analyzed the Medicare 5% sample file by race and ethnicity for both T1D and T2D for the years 2012–2013. Although nHWs accounted for the majority of this population, the prevalence of T1D and T2D was higher for Hispanics for both types of diabetes. Our economic findings related to this higher prevalence show that a diagnosis of diabetes adds significantly to the already increased costs for older individuals and Hispanics in the US population. Additionally, Hispanics tended to use more acute care resources, also, resulting in longer hospital stays. This highlights an important disparity in the use of health care resources across the two populations and may provide an opportunity for new approaches toward more cost-effective use of health care resources.

The health economic burden for Hispanic seniors with diabetes reported here is notable, but unsurprising. In the United States, ∼1 in 9 adults has diabetes; however, the prevalence of both diagnosed and undiagnosed diabetes is nearly twice as high among Hispanic adults of Mexican origin than nHW adults.^27^ Furthermore, Hispanics are also disproportionately impacted by complications of diabetes.^28^ The reasons for the excess burden of diabetes in Hispanic adults include confounding factors such as acculturation, age, and socioeconomic status in addition to much-studied biological risk factors such as obesity and high blood pressure.^29–32^ In the Medicare population, age per se also appears to add significantly to the health economic burden for individuals with diabetes beyond the costs of common microvascular and macrovascular complications.^33^ This may be relevant given our finding that in this cohort, the average age of Hispanics with diabetes was significantly higher compared with nHWs and could have added to the cost burden. As a corollary, some of the differences seen here could be attributed to the age bias of the sample and may not hold up when applied to the entire US population.

At all ages, rates of screening for complications of diabetes have been reported to be lower for Hispanics.^31^ Surprisingly, in this older population, we found that rates of screening for diabetes-related complications, HbA_1c_, and lipid assessments were performed more often in Hispanic seniors with diabetes than their nHW counterparts. One possible explanation for this finding could be increased proactivity among clinicians once this population is diagnosed with diabetes, and perhaps not reflective of screening and prevention rates before diagnosis. Indeed, the positive effects of insurance coverage on health outcomes for adults with long-term conditions such as diabetes include greater use of health services and improved health outcomes, including disease control.^34^ Thus, these findings are consistent with recent research indicating care improvement after diagnosis and suggest that access to Medicare further enables screening for complications as well as advanced treatment.

Fewer studies have been conducted on the complications of diabetes, and these generally suggest that the rates of complications of diabetes vary by type and by racial and ethnic minority groups.^35–38^ Consistent with the present study, another has reported that rates of early- and end-stage kidney disease are up to 2.5 times higher among Hispanics compared with nHWs.^39^ Despite a higher prevalence of obesity and risk factors for cardiovascular disease in Hispanics, some studies have suggested that Hispanics may have a lower risk of cardiovascular-related mortality (the Hispanic paradox).^40^ However, as this is not a consistent finding, cardiovascular disease in the Hispanic population needs further study.^41–42^

### Limitations

There are a number of important limitations to this study. The Medicare 5% sample applies only to seniors aged 65 years and above and therefore the results may not be applicable to younger age groups with diabetes. The sample size of Hispanic seniors with diabetes was also relatively small, limiting the applicability of findings. Furthermore, the T1D cohort is relatively small and the dataset does not detail the criteria for diagnosis of T1D versus other forms of diabetes. For the purposes of this study, CMS-approved ICD-9 diagnosis codes for T1D and T2D were used, which could have resulted in coding misclassifications. In addition, given the negative impact of a diagnosis of T1D at a young age on long-term survival,^43^ this cohort is unlikely to be representative of T1D in general. The Medicare 5% File does not contain outpatient prescription drug data, with only payments for inpatient medications included in the file.

There are also differences in the distribution of racial and ethnic groups between the national census and the Medicare 5% File. As per the national census, Hispanics comprised almost 8% of the population aged ≥65 years; however, in the Medicare 5% File, Hispanics comprised only 1.7% of all racial and ethnic groups. This could be due to varying definitions of race and ethnicity among the US census versus Medicare.

## Conclusion

Analysis of data from the Medicare 5% File showed that a diagnosis of diabetes in older Americans adds significantly to the cost of health care, and the prevalence of diabetes appears to be higher for Hispanics than for nHWs. Resource utilization, including hospital admissions and length of stay, was higher in Hispanics with diabetes, resulting in higher health care costs for this growing minority. While the disproportionate use of health care resources could be attributed to a variety of factors, the resulting spending for the health care system underlines the need for new approaches to diabetes care. Considering the high burden of chronic disease in Hispanics and the exponential growth in older US and Hispanic populations, this call for action to our health care providers is even more imperative.

## Abbreviations Used

CMS: Centers for Medicare & Medicaid Services
ESRD: end-stage renal disease
HbA1c: glycated hemoglobin
HCC: hierarchical condition category
ICD: International Classification of Diseases
LDL: low-density lipoprotein
nHW: non-Hispanic white
PMPM: per member per month
T1D: type 1 diabetes
T2D: type 2 diabetes
